# Advancing Sustainability in Modern Polymer Processing: Strategies for Waste Resource Recovery and Circular Economy Integration

**DOI:** 10.3390/polym17040522

**Published:** 2025-02-17

**Authors:** Ionut-Cristian Radu, Andreea-Mihaela Vadureanu, Derniza-Elena Cozorici, Erika Blanzeanu, Catalin Zaharia

**Affiliations:** Advanced Polymer Materials Group, Department of Bioresources and Polymer Science, Faculty of Chemical Engineering and Biotechnology, National University of Science and Technology POLITEHNICA Bucharest, 1-7 Gh. Polizu Street, 011061 Bucharest, Romania; ionut_cristian.radu@upb.ro (I.-C.R.); andreea.vadureanu@upb.ro (A.-M.V.); derniza.cozorici@upb.ro (D.-E.C.); erika.blanzeanu@upb.ro (E.B.)

**Keywords:** sustainable polymeric materials, circular economy, polymer processing, sustainable development, waste reduction

## Abstract

By the late 1970s, plastics had emerged as the most widely used materials globally. The discovery, development, and processing of diverse polymeric materials have profoundly shaped modern life and driven the expansion of numerous industries. Given the widespread interest in the utilization of these materials, it has become increasingly imperative to design their life cycles from the outset. This approach aims to maximize their utility while minimizing their environmental footprint. This review aims to identify and analyze the key challenges in polymer processing applicable to both additive and formative manufacturing methods, emphasizing the relationship between processing and recycling within the framework of sustainability. Modern polymer processing techniques play a crucial role in enhancing the sustainability of polymer products by improving recycling potential (with consideration of polymer type, source, and additives), cost-effectiveness, carbon footprint, and key properties such as durability, lifespan, performance, and environmental impact. It will also explore the concept of the circular economy and its integration into modern processing methods, including extrusion, injection molding, and 3D printing. Additionally, current polymer recycling methods are analyzed with respect to their effectiveness, sustainability, and compatibility with the original materials. Moreover, the discussion emphasizes the benefits of a circular economy compared to a linear one by exploring the concepts of closed-loop and open-loop systems, along with their diverse applications depending on the material and the initial processing method employed. To ensure that humanity continues to benefit from polymer materials while striving for a waste-free environment, it is essential to integrate the principles of sustainable development from the very beginning.

## 1. Introduction

The increasing environmental concerns surrounding plastic waste and the increasing consumption of non-renewable resources have underscored the urgent need for research and development of bio-based polymer materials as sustainable alternatives to traditional petroleum-based polymers. Although the exploration of polymer development has been ongoing for over a century, the focus has primarily been on discovering new materials that offer improved properties, suitability for innovative applications, simplified processing, or cost efficiency. In recent decades, however, significant challenges have emerged regarding the environmental impact, sustainability, and recycling options for these materials. This review aims to outline and discuss the key processing challenges associated with both additive and formative manufacturing techniques, with a particular focus on the interaction between processing methods and recycling within the framework of sustainability. Modern polymer processing methods play a vital role in promoting the sustainability of polymer products. This influence comprises factors such as recycling potential—considering the polymer type, its source, and any additives used—as well as cost-effectiveness, carbon emissions, and essential characteristics like durability, lifespan, performance, and environmental impact [1,2,3,4].

The global concern surrounding the sustainability of polymeric materials is a shared responsibility that requires concerted efforts. There are opportunities to reduce or eliminate the consumption and production of certain plastics while simultaneously developing more environmentally friendly materials. In 2024, South Korea hosted representatives from 175 nations to establish the first-ever global treaty focused on reducing plastic pollution. Additionally, numerous countries and cities have implemented policies to minimize the use of non-essential plastics, such as single-use items and unsustainable materials, and to enhance plastic waste management practices [3,5,6]. Not all plastics possess the same characteristics, applications, costs, sources, recyclability, or consumption patterns, highlighting the need for targeted strategies in both reducing plastic use and promoting the development of sustainable alternatives [7,8,9].

**Figure 1:**
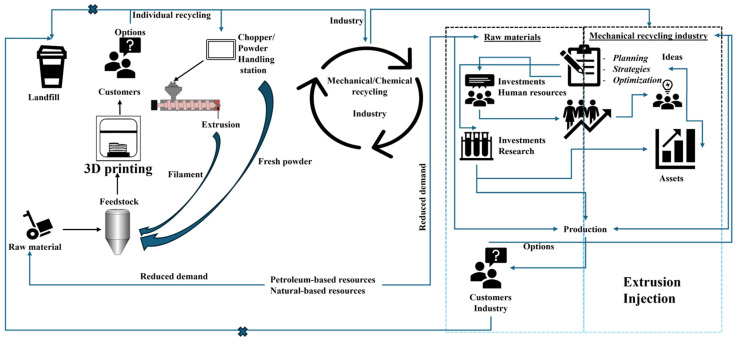
A holistic approach illustrating the relationship between modern polymer processing techniques, recycling strategies, and circular economy integration to advance sustainability.

Polymeric materials are likely capable of being recycled and reused multiple times, offering significant potential for industrial recycling through mechanical processes. Furthermore, high-value secondary raw materials are more sustainable than primary raw materials, as they typically have a lower environmental impact. The production of new polymeric materials is not only more expensive but also generates a significant carbon footprint due to various processes involved, such as monomer production, polymer synthesis, and material processing. On the other hand, producing high-value secondary raw materials poses challenges, as mechanical recycling technologies often struggle to accommodate the diverse range of polymeric materials and processing methods. The selection of processing methods, polymer types, and additives is primarily driven by the intended application, often with little consideration for recycling feasibility. A key challenge now is to ensure that ongoing technological advancements in processing are compatible with existing recycling infrastructures. The recycling sector faces difficulties in accommodating the various polymeric materials generated by different industrial processing methods. Therefore, collaborative advancement is essential between industrial recycling and processing infrastructure. This raises a significant question: can the processing industry adapt to manufacture polymeric materials with a sustainable focus? (Figure 1).

To date, the processing industry has demonstrated its readiness for the development and production of polymeric materials that meet application requirements. It is important to note that processed polymeric materials often include various types of additives that enhance processing and properties, yet this can reduce their recyclability potential. The influence of additives should be evaluated alongside their necessity, concentration, and the effective interaction between polymer–additive or additive–additive combinations. The primary objective is to enhance and tailor the design and development processes of polymeric materials, taking into account factors such as application, properties, durability, reusability, recyclability potential, environmental impact, and life-cycle costs. Moving forward, this optimization can help clarify the actual effectiveness of an additive, identify the minimum optimal dosage, determine the appropriate processing techniques, assess compatibility with specific polymers, and define the most suitable form of the additive, such as powder, particles, flakes, or masterbatch. These insights are crucial for advancing a sustainable approach. Optimizing design and development processes is essential, as it can reveal the actual effectiveness of an additive, establish the minimum optimal quantity, identify the appropriate processing techniques, evaluate compatibility with specific polymers, and determine the most suitable form of the additive, whether as powder, particles, flakes, or masterbatch. These findings are essential for a sustainable optimization process. Initial real data must be sourced from processing, research, or practical experience to create viable information. Nevertheless, the processing industry frequently relies on equipment from manufacturers that do not offer open interfaces and data models, which impedes the development of databases and makes it challenging to integrate these devices into automation platforms. Even when such input data is available, the management of large data volumes in polymer engineering demands advancements in digitalization and automation.

The integration of artificial intelligence and machine learning strategies is transforming polymer processing and manufacturing, leading to the emergence of a new interdisciplinary field known as Polymer Informatics, which bridges polymer science and computer science. This interdisciplinary focus is essential for optimizing processes and managing data, driving significant advancements in the polymer manufacturing industry. Polymer informatics leverages data-driven techniques to analyze input data, facilitating more efficient development, design, and discovery of novel polymers. While the exploration of new polymers from unique monomers or combinations is not the primary focus of polymer processing, the development and design of polymeric materials remain central to the field. The integration of artificial intelligence is poised to revolutionize polymer processing techniques—whether through formative or additive manufacturing—across various sectors, including food, construction, transportation, medicine, cosmetics, and agriculture [10,11,12,13,14,15]. These advancements are further elaborated upon in each section of the review.

## 2. Formative Processing of Polymers

Formative polymer processing involves shaping polymeric materials into a desired form using methods such as molding (e.g., stamping, high-injection molding, thermoforming), vacuum forming, or extrusion through a die. These processing methods are favored in industry for mass production due to their ability to produce polymeric products at low cost, with high speed, and consistent quality. Compared to additive manufacturing, formative manufacturing is better suited for the large-scale production of polymeric parts with complex geometries, uniform quality, and appealing aesthetics. Similar to 3D printing, injection molding and extrusion manufacturing generate their own polymeric waste. It is essential for manufacturers to incorporate recycled materials into their plastic injection or extrusion processes to promote sustainability. Modern extrusion devices can feature integrated regrind systems, enabling a seamless flow of secondary raw materials back into the primary feed. Combining the precision of 3D printing with the scalability of extrusion and injection processes presents an innovative approach to manufacturing new categories of polymeric products. Efforts toward sustainability necessitate addressing the technological limitations of existing infrastructures through investments in digitalization (machine learning and polymer informatics), automation, and skilled human resources. These strategies enable greater precision, reduced waste, optimized use of additives, enhanced energy efficiency, programmable setups, and the development of toxin-free materials. The implementation of green practices in manufacturing operations is essential to ensure that production processes are both environmentally friendly and sustainable [16,17,18,19,20,21,22]. As previously mentioned, a comparison between injection molding and other polymer processing techniques, such as 3D printing and extrusion, reveals both advantages and limitations in terms of process sustainability. For example, unlike injection molding, direct ink writing (a 3d-printing technique) often involves the use of solvents, which can harm the environment and complicate the process. This makes it more time-consuming, energy-intensive, and complex due to the additional separation step required. Similarly, stereolithography relies on resins that are often toxic, and the finished objects typically require additional solvents, such as isopropanol, to remove excess resin. When comparing the tensile properties of the same material processed through fused deposition modeling and injection molding, injection molding generally produces better tensile strength and elongation at break, likely due to the higher density of the resulting products. The enhanced mechanical properties can contribute to a longer service life, while the lighter weight of the products can help reduce material waste and lower transportation costs [23].

### 2.1. Injection Molding

**Figure 2:**
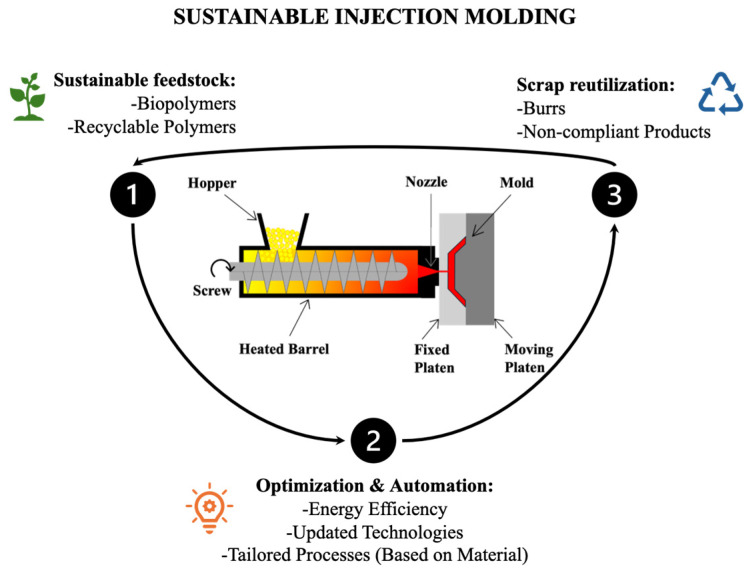
Schematic of an injection molding system with sustainability-focused enhancements.

Injection molding is a manufacturing technique used for shaping plastics and metals that originated in the 19th century. It was patented in the United States in 1872 by the Hyatt brothers, who used it to produce nitrocellulose, at that time known as pyroxylin [24,25,26,27]. Over time, injection molding became a relatively common manufacturing technique due to its ability to produce complex geometries quickly. To appreciate its significance in modern manufacturing, it is essential to understand the injection molding process, which explains its widespread use and efficiency in producing high-quality, intricate designs. The injection molding process, illustrated in Figure 2, involves melting and homogenizing the material in a plasticating unit before injecting it into a heated mold under high pressure. A screw-driven system, powered by either electric or hydraulic drives, facilitates material transport, melting, and accumulation prior to injection. After the mold is filled, the material solidifies—through cooling for thermoplastics or curing for thermosets—and the finished part is automatically ejected. Consistent heating along the barrel and nozzle ensures uniform melting and prevents blockages [28,29,30,31].

Injection molds typically consist of a stationary and a moving part that form the desired shape when closed. Multi-cavity designs enable the simultaneous production of multiple parts. A clamping unit applies force exceeding the injection pressure to maintain mold integrity during the process, while an integrated cooling system facilitates material solidification or reduces the part’s temperature before ejection [29,32]. Along with process parameters and mold design, the materials used in injection molding play a critical role. In addition to the polymer, the material fed into the hopper typically includes various additives, such as fillers to reduce the cost of the final product, reinforcing agents to enhance mechanical properties, plasticizers to improve processability, fibers to minimize object shrinkage, and lubricants to facilitate easier ejection of the finished object [29,32,33].

This process of combining a polymeric matrix with additives is known as compounding and results in a complex material system that is designed to improve various properties, such as reducing costs and enhancing mechanical strength and processability [30,34]. The compounding step can be carried out either prior to injection molding in a separate extruder or integrated directly within the injection molding process [25,35]. The fundamental principles of injection molding, as outlined above, form the basis for exploring its diverse applications across various industries.

Injection molding finds extensive application in various industries today. In the cosmetic and pharmaceutical sectors, it is widely used for packaging, while its potential in the biomedical field is being explored for creating scaffolds and microneedles [29,36,37,38]. On an industrial scale, one may emphasize the importance of this manufacturing technique in producing laboratory equipment, such as test tubes, Petri dishes, and microplates, as well as components for industries including aerospace, automotive, electronics, and consumer products [39]. In the medical field, injection molding is employed for producing medical disposables, devices, packaging, instruments, and plastic components [39,40,41,42,43]. Additionally, it is used in agriculture for irrigation parts and in the sports industry [40,44,45,46].

The versatility of this technique makes it highly advantageous across various industries. By modifying the mold geometry, a wide range of shapes can be produced with good accuracy and at high speed. However, the primary limitation of injection molding lies in the high cost of the molds, which restricts its use to applications that involve large production volumes [17,47,48]. Despite this limitation, the versatility of injection molding across industries has been further enhanced by continuous advancements in technology, which are discussed in the subsequent section. These advancements are mandatory at industry level from the economical and sustainable points of view. Therefore, injection molding has been adjusted to meet customer needs over the time. For example, companies such as Evco, HiTech, Protolabs, and Seaway Plastics Engineering offer various technological alternatives for injection molding. These include overmolding, where a thermoplastic material is molded over another to form a single object; gas-assisted injection molding, used to create hollow objects; and insert molding, where the polymer melt is molded around an insert piece, encapsulating it. Arburg, a leading manufacturer of high-quality machines for plastics processing, conducted a study to calculate the carbon footprint associated with the production of injection molding machines. The study considered factors such as raw materials used (including plastic, electronic, and metal components) and electricity consumption. According to Arburg, only 5% of the carbon footprint of an injection molding machine is attributed to its production, while a substantial 95% is associated with its usage [49]. Therefore, it is important to focus on using injection molding equipment more sustainably, as addressing environmental challenges is still vital despite technological advancements. The following section explores strategies for improving sustainability in injection molding, highlighting key approaches to reduce its environmental impact.

According to The PLASTEK Group, sustainability in injection molding involves optimizing the process to minimize energy and material consumption, ultimately reducing the carbon footprint of each manufactured object [50]. Figure 1 outlines key concepts to consider for achieving sustainability in injection molding processes.

In addition to The Plastek Group’s definition of sustainability, optimizing the use of time and human resources is crucial for improving the sustainability of the injection molding process. Automation has significantly advanced this goal by streamlining tasks such as mold ejection and material dosing, reducing the need for manual labor and minimizing human error, as seen with the use of industrial silos coupled with automatic scales and modern dosing and mixing solutions [51]. Building on these advancements, artificial intelligence and simulations offer further potential to optimize process parameters, which helps reduce energy waste and scrap when working with new materials. While this approach enhances accuracy and sustainability, its main challenge lies in balancing predictive precision with cost and time efficiency [52].

Research has been carried out to identify the major energy consumers in an injection molding system. For example, an analysis of the injection molding process using high-impact grade ABS polymer was conducted to identify the process stages and machine components that consumed the most energy, with the aim of enhancing sustainability and efficiency. The study examined five process parameters: melt temperature, mold temperature, holding time, holding pressure, and cooling time. Results showed that the cooling stage was the largest energy consumer, accounting for approximately 41% of the total energy usage, compared to 21% for plasticizing and 14% for holding. On the equipment side, the temperature control unit and chiller pumps were responsible for approximately 50% of the total energy consumption, while the injection molding machine itself accounted for 30%, and the chiller contributed the remaining 20% [53]. To optimize energy usage, focus should be first of all put on designing equipment with lower energy consumption, while users must implement effective optimization and maintenance strategies, emphasizing preventive maintenance. Additional waste may arise from outdated equipment that consumes more energy and generates defects like burrs, as well as from insufficient maintenance or excessive wear. By targeting key parameters such as screw rotation speed, mold closing movement, injection pressure, and mold opening speed, users can achieve lower specific energy consumption and shorter cycle times, leading to substantial energy savings and improved production efficiency. End-users of injection molding machines should engage in regular research and development to streamline the process for each step and adapt it to different materials or material combinations [54,55,56,57].

In addition to the diverse range of injection molding technologies, numerous sustainable materials have been developed to meet specific requirements, including those aligned with sustainability goals. The focus is on polymers produced through greener processes, characterized by low environmental factors—a metric that evaluates the efficiency of chemical processes based on waste generation [58]. Beyond the E-factor, these sustainable production routes should also consider waste toxicity, energy and water consumption. These sustainable alternatives of raw materials refer to biomass-derived biopolymers, compostable polymers, and recyclable polymers, which can be reprocessed into new products once they have reached the end of their lifecycle.

Examples of biomass-derived polymers used in the literature as raw materials for injection molding include rice bran [59,60,61], and zein [62,63,64]. Natural reinforcements commonly employed are bamboo fiber [65,66], hemp fiber [67], and banana fiber [68], while glycerol is widely used as plasticizer [60,62].

The targeted industry is actively working to provide greener material options suitable for injection molding. In this direction, Table 1 highlights commercially available materials designed as more sustainable alternatives to traditional petroleum-based polymers used in the process.

Different strategies emerge in the pursuit of more sustainable material options. First, there is a strong shift toward the use of renewable and bio-based materials. Additionally, the integration of natural fibers into polymeric matrices helps reduce fossil-based plastic content. Another key focus is compostability and biodegradability, achieved through the use of biodegradable polymers.

As previously mentioned, research in the field of injection molding is primarily focused on the addition of fillers or reinforcements, particularly those that are readily available in nature or derived from various types of waste. Regardless of their source, natural fibers can be considered composite materials due to their complexity [28]. The incorporation of fillers or reinforcements into the polymer matrix can impact the recyclability of the final product and complicate its integration into the circular economy. For instance, this can create challenges in the chemical recycling of polymers, as separating the additives often requires additional, complex steps. For the recycling process to be viable, the separated fillers or reinforcing agents must exhibit properties comparable to those of virgin materials. A notable example is the recycling of carbon fibers from thermoset matrices, widely used as reinforcing agents in the automotive industry due to their properties closely resembling those of virgin materials [77]. On the other hand, in thermoplastic matrices, when the material is chopped and reintroduced into a molding machine, degradation may occur depending on the nature of the filler. For instance, bamboo fiber-reinforced PLA shows reduced thermal stability after recycling [65]. Achieving a sustainable injection molding process requires adopting a broader perspective that considers material sourcing, processing, product lifecycle, and recycling opportunities. Research indicates that the addition of fibers can reduce injection pressure, thereby lowering energy consumption. However, maintaining a balance is essential—if the injection pressure is too low, the mold may not fill properly [78]. While sustainable materials are important, managing and reducing scrap is equally vital for a sustainable manufacturing process. This includes addressing waste and burr management, optimizing disposal methods, and minimizing transportation distances to reduce fuel consumption and the product’s overall carbon footprint [79,80].

Additionally, besides burrs and defective lots, another common source of scrap in injection molding arises during raw material changeovers, when the equipment is cleaned with the new material until it fully replaces the previous one. This process generates a byproduct known as purge, a mixture of polymers with varying characteristics and sizes, which is typically disposed of, further contributing to waste [81]. In terms of sustainability, injection molding has an advantage over extrusion due to its superior ability to control the final shape, resulting in fewer defects, reduced scrap, and a more sustainable processing technique. While the cyclic and discontinuous nature of injection molding might limit throughput and cause downtime, using multiple molds, such as in multishot equipment, can help mitigate these drawbacks. However, there are still other factors to consider, such as inconsistencies in material properties between cycles, high energy consumption during each cycle (caused by heating and cooling), the cycle time’s dependence on the polymer cooling phase, and burrs generation. Although waste materials, such as burrs or defective parts, can be reprocessed into new objects to conserve material, this reprocessing requires additional energy, which diminishes the overall sustainability of the process.

### 2.2. Extrusion Molding

Extrusion is among the most widely used manufacturing processes for polymers. As a well-established forming method in the plastics industry, it is employed to produce a diverse range of polymer-based products for various markets. The extruded plastics market is segmented based on the materials used, including commonly employed thermoplastic polymers such as polyvinyl chloride (PVC), polyethylene (PE), polypropylene (PP), and polystyrene (PS). It is further classified based on end-use industries, including packaging, automotive, construction, electronics, medical, and consumer goods. Moreover, the market is segmented by extrusion technology and geographical regions. Extrusion involves forcing molten polymer through a die to produce a continuous profile with a specific cross-sectional shape. A polymer extrusion system comprises a drive unit to rotate the screw, a hopper for material feeding, precise heating and cooling systems, and a die for shaping the molten polymer. In a complete extrusion line, additional components such as cooling tanks, pullers, and cutters are integrated based on product specifications. The screw, the core component of the extruder, facilitates material transport, melting, homogeneous mixing, compression, and pressure generation for die extrusion [82,83,84,85]. The elevated temperatures reached during the melting process, combined with the polymers’ residence time in the barrel, can lead to polymer degradation, which directly impacts the properties of the final extrudate. Such degradation compromises the performance of the extrudate in service by reducing its molecular weight and altering its mechanical properties [86,87,88].

The screw design is another important factor that influences the efficiency and quality of the polymer processing. The design and selection of screws are guided by key factors such as the polymer being processed, the required throughput rate, the consistency and uniformity of the polymer melt during the entire process, die pressure development, the needed mixing efficiency, and energy consumption [89,90]. The efficiency of the extrusion process is directly affected by the characteristics of the screw. Both the functionality and design of the screw play a critical role in determining the efficiency and quality of polymer processing during extrusion. For a conventional screw, critical parameters include its diameter, relative length, and the number and length of its functional zones. Key factors also include the compression ratio, defined as the ratio of the channel depth in the feed section to that in the metering section, as well as the geometry of the screw channel. The geometry of the screw channel specifically refers to features such as the pitch—the distance between two successive flights, the flight width, the channel depth—the distance from the top of the flight to the root, and the channel width—the space between adjacent flights. All these elements are critical in determining the performance of the screw [91,92,93,94,95,96,97,98,99]. Therefore, melt temperature profiles in polymer extrusion are heavily influenced by the properties of the polymer, the geometry of the screw, and other process parameters, such as the screw’s rotation rate.

This outlines the fundamental operation of a single-screw extruder, which remains the most widely used equipment in polymer processing within the industry. However, polymer extrusion technology has advanced significantly over the years, with key innovations driving enhancements in both efficiency and product quality. The evolution of extrusion technology, from single-screw extruders to multi-screw designs, starting with twin-screw extruders and progressing to triple-screw, quad-screw, octa-screw, and planetary extruders, has been marked by significant efforts to enhance melting and mixing processes for complex polymer blends and compounds. These advancements have been driven by progress in technology and materials development, as well as a deeper understanding of polymer science, especially in terms of thermal and melt flow behavior [100,101,102].

In addition to screw-based extrusion processes, there are screwless extruders, which rely on mechanisms such as drums or disks to transport the polymer within the barrel. These systems are classified under the broader category of continuous extrusion. Additionally, ram extruders, also known as plunger extruders, operate in a discontinuous manner, providing a distinct method for polymer processing [103].

In the pursuit of advancements in extrusion techniques, several innovative methods can be identified. Coextrusion, for example, involves extruding two or more polymeric materials through a single die to create multilayer products with integrated layers that offer distinct functionalities [104,105]. Foam extrusion, on the other hand, involves incorporating a foaming agent, such as nitrogen or carbon dioxide, into molten polymer, creating a cellular structure. This process results in lightweight materials with enhanced insulation, sorption properties, and improved impact resistance [106,107]. Furthermore, the integration of extrusion with 3D-printing technology enables the creation of customized and intricate structures. These combined approaches are widely recognized for their capability to produce complex blends and composites, enhancing both structural integrity and functionality.

Computer-controlled processes and automation are essential to modern plastic extrusion today, ensuring enhanced precision and consistency. In addition, the advancements in sensing technologies for process monitoring enabled continuous tracking and adjustment of critical parameters such as melt temperature, pressure, and viscosity. By maintaining these parameters within optimal ranges, employing different monitoring techniques ensures consistent product quality, facilitates the early detection of process fluctuations, and minimizes variations in the extruded product quality [108]. As modern extrusion techniques continue to expand, the plastics market grows alongside them, intensifying environmental concerns due to persistent recycling inefficiencies. This, in turn, increases sustainability pressures and the demand for stricter regulations and compliance. The demand for sustainable polymeric materials in extrusion processes is rapidly increasing, driven by environmental concerns and the growing emphasis on a circular economy in polymer processing. This shift has emerged as a response to the challenges posed by disposable polymer-based products and waste management issues.

#### 2.2.1. Sustainable Practices and Strategies in Polymer Extrusion

**Figure 3:**
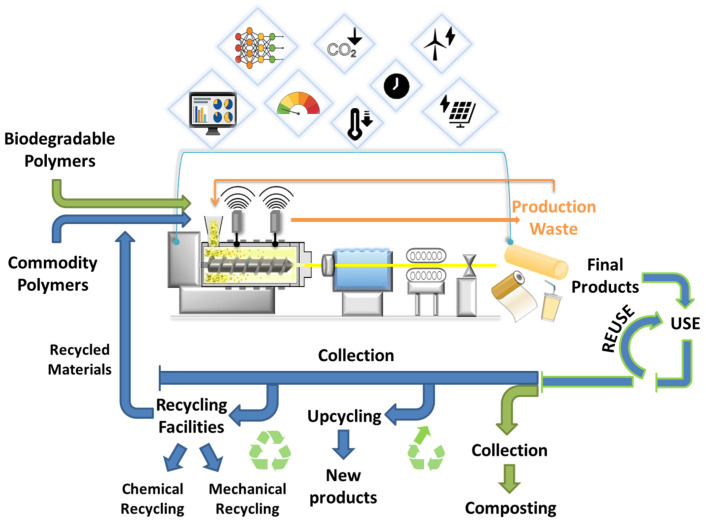
Sustainability in extrusion: optimization and circularity.

Sustainability can be implemented at every step of the extrusion process. It starts with selecting polymers based on their degradability and recyclability, which directly impact the environmental fate of the extrudate products at the end of their life cycle. At the same time, optimizing the parameters of the process can help minimize waste and reduce energy consumption, further enhancing sustainability. This can be achieved by first fine-tuning the design, configuration, and maintenance of the components of the extrusion machines, such as the screws, barrels, and dies, and then sustaining it through continuous monitoring, further enhanced by simulation and modeling tools [109]. Figure 3 describes the sustainability measures in extrusion, focusing on process optimization, waste reduction, and circular material flow.

The development of new materials and the optimization of processing parameters are both crucial for promoting sustainability in the extrusion process. On one hand, the development of biodegradable and easily recyclable alternatives can help mitigate the environmental challenges posed by commodity polymers. These alternatives offer more sustainable disposal options, particularly for single-use extruded products, by reducing waste accumulation and its environmental impact. Additionally, they support the promotion of a circular lifecycle, where materials are recycled and reused. Continuous efforts are being made in this direction to overcome the existing challenges in terms of production costs and product quality, compared to the well-established polymers that have been used so far [110,111,112]. On the other hand, optimizing processing parameters can significantly reduce energy consumption and enhance the quality of extrudate products, thereby improving the performance and lifecycle of existing materials. Sustainability improvements through processing optimization alone are limited by the characteristics of the polymer used, as if the material is not degradable or recyclable, optimizing the process has minimal impact on the product’s end-of-life. Therefore, employing both innovation in material development and processing optimization is essential for achieving overall extrusion process sustainability.

#### 2.2.2. Sustainable Material Choices

In the sustainability context, selecting materials based on their resource origin, degradability, and recyclability significantly influences their life cycle impact, directly affecting waste generation after the extrusion process.

The literature extensively explores the use of natural polymers as alternative feedstocks to petroleum-based polymers commonly used in the extrusion sector, considering their renewable nature and degradation characteristics. At the same time, the development of biodegradable polymeric materials aimed at providing eco-friendly solutions that meet industry and market demands is also noteworthy. Examples include the expansion of polymer blends and composites [113,114,115,116,117,118,119], as well as enhanced biodegradation profiles, where polymers are designed to degrade upon exposure to ambient stimuli after use [118,120,121,122].

In addition, waste valorization is another promising approach being explored to develop sustainable materials for extrusion. This includes utilizing post-consumer recycled plastics [123,124,125] as well as valorizing biomass and agricultural by-products [126,127,128,129,130,131].

A review of the material portfolios of plastic extrusion developers and manufacturers in the polymer processing industry highlights various approaches to incorporating sustainable materials in line with circular economy principles. These include integrating raw materials derived from renewable resources, reinforcing commonly used or recycled polymers with natural fibers such as ground wood, coconut shells, or wood byproducts [132,133,134,135], and utilizing polymers that can be processed at lower melt temperatures [136]. These efforts aim to reduce the carbon footprint while maintaining the quality of their products.

Table 2 lists commercially available green material solutions, highlighting the adoption of hybrid materials derived from renewable and bio-based resources, specifically designed to degrade under various composting conditions.

While the shift towards sustainable polymers in extrusion offers numerous advantages, challenges remain in implementing these strategies within an industrial context. These challenges include the limited ability to fully replace synthetic polymers, the need for industries to adapt to new materials and technologies, and the higher production costs.

#### 2.2.3. Sustainable Process in Extrusion

Energy consumption in the extrusion process is an additional concern due to its energy-intensive nature, which impacts both environmental sustainability and operational costs. By analyzing the energy demand of extrusion systems and identifying potential energy losses throughout the process, the influence of various factors on extruder energy consumption has been studied over the years. Identifying key energy consumption factors aims to optimize the process, balancing energy savings with maintaining the quality of the extrudate product. At the extrusion machine level, the drive motor is the largest energy consumer in the extrusion process, followed by the heating and cooling systems, as well as the compressors. Additionally, a significant amount of heat energy is lost during the extrusion process, highlighting the need for waste heat recovery and utilization methods [109,145]. One such method is described in the study by Zauner et al., which presents an energy-efficient factory concept. This concept incorporates an insulated hot water bath, a high-temperature heat pump, and latent heat storage to recover and reuse waste heat for industrial processes and space heating, demonstrated in a real factory setting [146].

At another level, the relationship between the extruder’s total energy consumption and process parameters reveals that barrel heating temperature negatively affects energy efficiency. In contrast, optimizing screw speed enhances mechanical work heat, thereby reducing the energy required for polymer heating [147,148,149]. Additionally, the relationship between power consumption and polymer properties reveals that rheological characteristics significantly influence energy use, as different polymers have varying energy requirements during extrusion [149]. In this context, modeling and numerical simulation methods can be used to optimize the extrusion process by analyzing the behavior of polymeric materials under various conditions or by simulating variations in operational parameters. These approaches can help predict polymer melt flow or thermal dynamics, thereby reducing environmental impact by minimizing unnecessary energy consumption [150,151,152,153,154]. Applying these strategies to the design of efficient extrusion dies is another area of exploration, as dies significantly influence the extrusion process, affecting both product quality and process efficiency [155,156,157]. Therefore, implementing energy-efficient machines and optimizing processing conditions can lead to significant energy savings, reducing the energy footprint of the polymer processing. In this direction, trends in polymer extrusion energy reduction focus on advancements in extruder machine design, incorporating advanced process monitoring and control systems, along with improvements in computational modeling capabilities. Additionally, efforts are directed toward waste heat energy recovery and the selection of more sustainable materials, such as polymers with lower melt temperatures [109].

#### 2.2.4. Principles of Sustainable Practices

Environmental initiatives in polymer extrusion show that manufacturers are increasingly adopting eco-friendly practices, such as using recycled materials, optimizing energy consumption, and designing products from the outset for recyclability. In this context applying Life Cycle Assessments (LCA) to better understand the environmental impacts of products, such as greenhouse gas emissions, and identifying opportunities to optimize production processes or materials efficiency, is also a sustainable practice that helps reduce the product’s environmental footprint. To further promote sustainability, green supply chain management is key in embedding eco-friendly practices throughout the supply chain [158,159,160].

Recycling as a sustainable practice in polymer processing extrusion is crucial for reducing waste and carbon emissions. In the context of a circular economy, designing products for recyclability at the end of their life to be remade into the same part is a key practice. This approach is exemplified by PolyVisions Inc., which uses post-consumer polyethylene terephthalate (PET) to produce graft-modified PET [161]. Other recycled materials that can be further extruded include polyvinyl chloride (PVC), low-density polyethylene (LDPE), and high-density polyethylene (HDPE) [162,163].

## 3. Additive Manufacturing of Polymers

**Figure 4:**
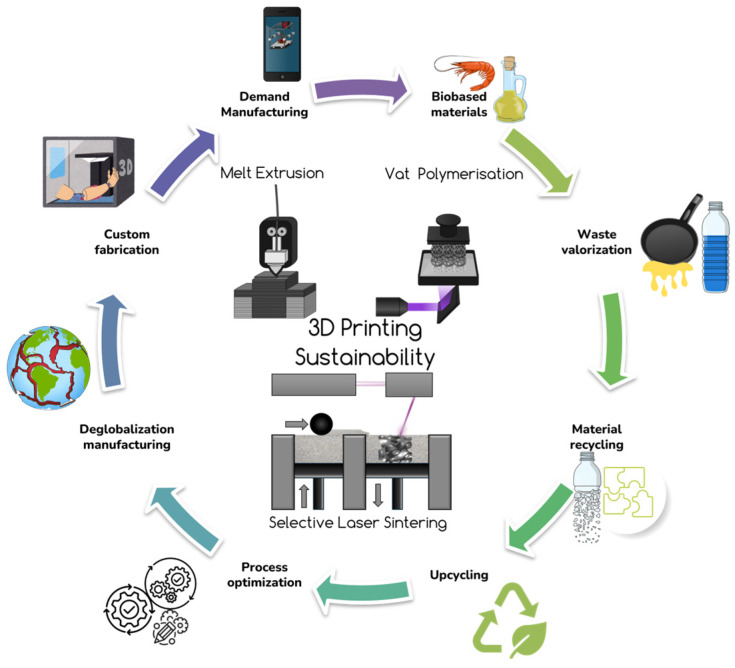
Strategic approaches to enhancing sustainability through various 3D-printing techniques.

Additive manufacturing is an emerging technology with tremendous potential for the industrial sector and domestic applications. The idea of translating virtual solid model data into physical models via quick and easy processing at home encouraged buying 3d-printer devices all over the world. This approach brought development of new types of raw materials (thermoplastic polymers, thermoplastic elastomers, powders, polymer precursors, etc.), an up-scaling production and new plastic pollution. This emergent technology came also with huge environmental responsibilities for the 3D printer and raw materials producers, and for the domestic and various economic sector users. The 3D printer feedstock can be solved by newly bought raw materials or via recycled secondary raw materials. However, the collection and transportation have an environmental impact increasing the greenhouse gas emission and costs. The approach to waste management can evolve from a centralized polymer recycling model to a more distributed system, where the responsibility of recycling is placed on the owners, including both households and commercial entities. In this context, 3D printer manufacturers must adapt their equipment to integrate plastic extruders into domestic waste and follow specific procedures to enable the use of recycled materials as feedstock. This strategy is particularly suited for extrusion-based methods, which utilize thermoplastic polymers and thermoplastic elastomer filaments, in contrast to vat-photopolymerization and powder bed techniques. The discussion will center on exposing the methodologies employed in 3D printing with polymers and polymer composites: material extrusion (ME), vat photopolymerization (VP), powder bed fusion (PBF). The process of photopolymerization in 3D printing results in crosslinked 3D structures that cannot be mechanically recycled due to their loss of chemical reactivity. Although chemical recycling could serve as an alternative, it necessitates that the polymer precursors are initially formulated with recycling potential. The polythiourethane chemistry supports various reversible click reactions between polythiols and polyisocyanates in the presence of organic bases [164]. Furthermore, acrylic monomers integrated with dynamic disulfide bonds are compatible with commercial stereolithography 3d-printing technologies. Numerous bio-based resins are accessible for industrial and household applications, enabling high-resolution printing, breakdown, recycling, and reprinting in a nearly closed-loop manner. It is important to note that the chemical recycling process is limited to centralized recycling operations and cannot be effectively implemented through a distributed model. This process implies a variety of chemical reactions and the application of different materials, such as solvents, monomers, oligomers, and catalysts, which are not feasible for home or small business reproduction. In 2020, 18,500 tons of plastic were utilized through 3D printing; however, an exact estimate of the amount of waste generated, particularly from support structures and failed prints, remains unknown [165]. With the 3d-printing market growing at a rapid annual rate of approximately 25%, the need for sustainable 3d-printing materials has become increasingly critical to ensure the long-term viability and success of the industry [166,167]. Figure 4 provides an overview of the key strategies for utilizing various 3d-printing technologies to promote and enhance sustainability across various applications.

In order to highlight the environmental impact of 3D printing in the polymer processing domain, it is important to expose the main 3D-printing technologies specific to polymers.

### 3.1. Extrusion-Based Technology

The most common 3D-printing method is melt extrusion (ME), commonly known as fused deposition modeling (FDM) or filament fabrication (FFF). This technique builds objects by depositing melted filament in successive layers [168]. FDM technology is a popular method for processing thermoplastic polymers, offering advantages such as low cost, simple operation, and material compatibility. This technology is used in various applications, including concept modeling, decorative items, and functional parts. The adoption of FDM 3D-printing technology is progressively transitioning from research settings to industrial applications and showing promise for domestic use. During FDM 3D printing, thermoplastic filaments are heated to their melting point and extruded layer by layer through a heated nozzle, following a computer-controlled path. As the extruded material cools and hardens, it forms a 3D object with a desired structure [169,170,171,172].

Commercial filaments have been developed from various polymers, such as thermoplastic polyurethane (TPU), polyethylene terephthalate (PET), acrylonitrile butadiene styrene (ABS), polylactic acid (PLA), polyvinyl alcohol (PVA), polycarbonate (PC), polyamides (PA) and polypropylene (PP) [173,174,175]. Due to the rapid advancement of FDM 3D printing, the need to develop new materials derived from sustainable sources has become increasingly imperative. The development of fiber composite filaments for 3D printers has drawn more attention from researchers recently. For example, Pereira et al. [176] created a natural fiber filament for FDM by filling it with rice husk. The dehusking step of the rice-milling process produced the rice husks. Xie and his team [177] used PLA and wood flour to make a filament. The powdered wood was then sieved to create particles with a size range of 140 to 160 mesh. They used distilled water, glycerol, and tributyl citrate in the study. To create the filaments, 630 g of PLA and 270 g of wood flour were combined. In a previous study, our team obtained polylactic acid–chitosan composites, using two commercial chitosan powder and two chitosan extracts derived from shrimp head and shrimp shell from waste. The composite filaments were then prepared and 3D-printed into objects with a higher surface area-to-volume ratio [178]. The tendency is to use filaments made from recycled materials. The sustainability of the recycling process is largely dependent on how much energy is used. According to published data [179], recycling filament from 3D printing usually uses less energy than creating virgin filament. Utilizing filaments derived from recycled sources requires investigating the variations in the properties of items produced using virgin materials compared to those fabricated from recycled alternatives. Similar properties between these two types of materials can be obtained by optimizing the printing parameters. The study demonstrated that infill density and layer height significantly influence the compressive strength of recycled acrylonitrile styrene acrylate samples, achieving acceptable values [180]. By recycling PET bottles, researchers have managed to obtain filament suitable for 3D printing. Compared to PLA, using recycled PET (polyethylene terephthalate) plastic in 3D printing has a number of environmental advantages. It lowers greenhouse gas emissions by consuming less energy and carbon during manufacturing [181]. Another study focused on using PLA waste combined with virgin PLA filament to produce biocomposite-based recycled material for FDM 3D printing, yielding promising result [182]. Waste from electronic equipment can also be recycled to produce filament for 3D printing [183].

Table 3 presents an overview of commercially available filaments for FDM, highlighting sustainable alternatives for 3d-printing applications. The table highlights various filament types designed to minimize environmental impact through the incorporation of recycled materials, the use of bio-based sources, the promotion of compostability and biodegradability, and the integration of innovative additives such as algae and wood fibers.

FDM has numerous parameters that have important effect on the quality of printed object: nozzle temperature (NT), the infill raster density (ID), bed temperature (BT), raster deposition angle (RDA), fused filament printing speed (PS) and layer deposition thickness (LT). Examining the best parameters combination might be helpful in developing guidelines that support strategic decision-making for improving sustainability by consuming less resources [193]. This research [194] explored the influence of six printing parameters in Specific Printing Power (SPP), Energy Printing Consumption (EPC) and Specific Printing Energy (SPE) and concluded that while the ID primarily affects the SPP, printing speed and layer thickness have a major impact on EPC and SPE. According to another study [195], choosing the best 3d-printing parameters to reduce energy use can result in an energy consumption reduction of 48–72% when compared to the average or maximum energy used.

The sustainability of the circular economy can be further enhanced through 3D printing, which enables the repair of defective parts and extends their lifespan. Through the optimization of parameters like infill percentage set at 30% and roster angle 60%, researchers have established that 3d-printing repair offers a perfect equilibrium between structural integrity and material distribution efficiency, ensuring remarkable durability [196]. The application of artificial intelligence methodologies is driving significant advancements in additive manufacturing, including design optimization, improved process and quality control, accelerated materials discovery, and the streamlining of manufacturing operations. These technological developments are accelerating the implementation of additive manufacturing across multiple industrial sectors, thereby facilitating the creation of innovative products and more efficient manufacturing procedures. Upon the modification or expansion of the training datasets, AI solutions demonstrate adaptability to diverse technologies, materials, and multi-material 3D-printing processes [197]. Furthermore, biologically inspired design concepts are combined with practical engineering solutions through generative AI to create bioinspired materials. By using additive manufacturing (AM) and generative algorithms, researchers can explore complex biological phenomena and translate these insights into innovative 3D-printed designs [198].

### 3.2. Vat Polymerization Technology

Vat polymerization (VP) is a 3D-printing technique that uses photopolymerization to cure the liquid resin presented in a vat into a volumetric structure in a layer-by-layer manner. The resin curing process involves the use of an UV or visible light and a platform that gradually lowers the object as each layer is cured, having the benefits of efficient printing, higher accuracy and great resolution [199]. The photosensitive resin used in vat polymerization typically consists of an initiator, monomer, oligomer, and various additives. Its viscosity, a critical parameter for successful polymerization, must be carefully controlled within the range of 0.25 to 5 Pa·s. The curing process for (meth)acrylate resins involves radical chain-growth polymerization, whereas epoxy resins undergo polymerization through a step-growth process driven by a cationic mechanism [200]. Acrylate resins, which undergo free-radical polymerization, are the preferred photosensitive resins for vat polymerization technologies due to their high photopolymerization curing speed. The literature discusses multiple types of vat polymerization, though the best recognized and frequently employed ones are stereolithography (SLA) and digital light processing (DLP) [201]. The SLA printing methods use a laser beam that sweeps around polymerizing single lines of the ink until completing each layer. In SLA printing, ultraviolet light is projected in a series of patterns onto a vat containing liquid resin, enabling photo-crosslinking throughout the build volume. Second-generation SLA technology employs mask projection to cure entire layers of an object simultaneously [202,203]. DLP printing uses a projector equipped with a liquid crystal display (LCD) or a digital micromirror device (DMD) to project entire 2D layers onto the resin, resulting in significantly faster printing speeds compared to SLA. This technology has advanced to achieve resolutions ranging from 25 to 50 μm [201,202,204,205].

Regarding vat polymerization techniques, several approaches are being explored to meet sustainability goals, including enhanced recyclability, the increased use of bio-based materials and process optimization. Vat photopolymerization waste is particularly problematic because the materials used are usually thermosets that are not recyclable or reusable. E. Maines and colleagues investigated the mechanical recycling of 3D-printed objects through the cryomilling technique. The resulting powder was incorporated into fresh resin at concentrations of up to 30% by weight, and tests conducted on newly fabricated objects demonstrated promising properties [206]. In another study, a fully renewable lipoate-based photopolymer resin was developed, allowing for the 3D printing of high-resolution objects that can be efficiently disassembled and circularly reprinted [207].

Through the incorporation of Bond exchange reactions (BERs), acrylate-epoxy hybrid resin was obtained. By use of DLP 3D printing and a two-stage curing approach, exceptional mechanical properties were achieved. The utilization of a small-molecule-assisted BER technique could effectively break down the thermosetting printed components into soluble oligomers, capable of reassembling through reversible BERs. Hence, the recycled solution may be utilized in the subsequent printing cycle by eliminating surplus ethylene glycol and blending with new acrylate resins [208]. The research conducted by Alexa Kuenstler and his team revealed that the introduction of thioester groups can induce a reversion to the initial oligomers within the resin, enabling the reprinting of the mixture with great mechanical proprieties [209].

The methods and techniques outlined above were developed to recycle thermoset materials produced via vat polymerization, yielding promising results. Consequently, manufacturers of printable resins are encouraged to adopt these findings, enabling the recycling and reuse of printed materials and contributing to the establishment of a circular economy. There are currently several biobased formulations on the market for resins used in 3D printing. For example, Prusament biobased 60, which contains 60% of ingredients came from plant-based sources. eSUN’s PLA-based bio-resin is made using polylactic acid which is derived from corn—a renewable resource, according to the manufacturer. Although there are several biobased resin formulations on the market, the need to develop other biobased materials, possibly from waste, remains a goal for researchers [210,211,212,213]. Vegetable oils (Vos), also known as plant-based or natural oils, are derived from a variety of plant and tree sources. These oils are widely available across the world, thus presenting a sustainable alternative to chemical feedstocks. Fatty acids (linoleic, palmitic, oleic) constitute approximately 95% of their mass, with compositional variations dependent upon the plant oil origin (soybean, flaxseed, castor, palm, grapeseed and linseed). Epoxidation and acrylation are two examples of chemical modifications of VOs that have been thoroughly investigated, along with their application in 3D printing [214,215,216,217,218,219,220].

The role of filler additives is critical in polymer processing, and additive manufacturing is no exception. These additives can significantly enhance the properties of 3D-printed objects. To support sustainability efforts, an increasing number of fillers are being sourced from waste materials or derived from bio-based resources, contributing to a positive environmental impact [221]. Lignin nanoparticles were incorporated into acrylic resin, resulting in improved mechanical properties of 3D-printed objects compared to those produced with the control resin [222]. A high-performance, bio-based photosensitive resin for UV-curing 3D printing was developed using itaconic acid and bio-sourced diols. The incorporation of ZnCl₂ filler powder further enhances the mechanical properties of the resin, achieving a strength comparable to that of conventional acrylic photosensitive resins [223].

The primary ways in which SLA/DLP 3D-printing materials contribute to sustainability have been outlined. However, there remain areas for improvement, including minimizing solvent usage during production, eliminating photoinitiators, and developing one-pot, solvent-free formulations [224]. In this research, a straightforward method for optimizing parameters to minimize manufacturing time and material waste during 3D printing has been successfully implemented. This approach involves a two-stage processing strategy, starting with the printing of a flat specimen. This method significantly reduces printing time, eliminates the need for supports, and results in a partially cured specimen. Subsequently, the partially cured specimen is swiftly bent into the desired shape, followed by UV and thermal post-curing treatments to enhance the cross-link density and permanently fix the intended shape [225,226].

### 3.3. Powder Bed Fusion Technology

Powder bed fusion (PBF) includes selective laser sintering (SLS), a technique that uses high-powered lasers or electron beams to fuse polymer powder particles. As with other 3D-printing technologies, the laser selectively melts the powder at specific locations for each layer based on the design specifications. The quality of the final SLS print is highly dependent on processing parameters, particularly laser power and speed [227,228,229]. Materials used in PBF-based printing must be in powder form, yet only a limited number of methods are available for producing pure polymers as powders. One common approach is mechanical processing, which involves grinding and milling polymers into small pellets, often carried out in a cryogenic atmosphere to improve efficiency and maintain material integrity. Thermally induced phase separation plays a key role in the solution-based method. During this process, a temperature change causes the polymer solution to precipitate into microparticles. In contrast, the melt-based approach utilizes a coextrusion technique, where the polymer-containing melt undergoes emulsification. This emulsion is then cooled in a solvent, which dissolves the polymer matrix, leading to the formation of the desired powder. The spherical particles are recovered through filtration or sieving. The processing, mechanical, and functional properties of printed polymers can be significantly enhanced by blending them with ceramic, metallic, or carbon nanoparticles [230,231].

SLS has the potential to revolutionize the manufacturing industry, enabling the production of high-quality, customized parts with improved efficiency and sustainability [232]. Polyamide-11 (PA11) and polyamide-12 (P12), Polyetheretherketone (PEEK), polystyrene (PS), thermoplastic polyurethane (TPU), polypropylene (PP), and polycarbonate (PC) are the most commonly used polymers for SLS [233,234,235,236,237].

The use of biopolymers in SLS is necessary to prevent pollution caused by petroleum-based materials and to provide a wider range of feasible materials. For example, lignin is presented as an appropriate component for selective laser sintering (SLS) of polyamide-12 to lower costs while maintaining or increasing performance and processability [238]. Another biobased and biodegradable polymer, poly(3-hydroxybutyrate-co-3-hydroxyhexanoate), was successfully produced in powder form with a spherical shape and dimensions of approximately 100 μm. This polymer has also demonstrated compatibility with SLS technology for 3D printing [239]. Sisal fiber, a type of biomass derived from sustainable agricultural waste, was used to create a biobased material. The sisal fiber powder was blended with poly(ether sulfone) (PES) powder, resulting in a composite material suitable for 3D printing using SLS technology [240]. Additionally, a new biobased and biocompatible material for SLS has been developed using Poly(butylene succinate) (PBS). This material, produced through the emulsion solvent evaporation technique, is available in the form of microspherical powder particles. The printed models exhibited both excellent mechanical and biochemical properties, making them highly suitable for applications in tissue engineering [241]. As with other types of 3D printing, such as VP and FDM, there is a growing interest in developing new materials derived from waste to promote sustainability in SLS technology. A. Toncheva and her team developed polymer composites loaded with tire rubber (TR) waste. Two types of polymer matrices were used for this purpose: thermoplastic polyurethane (TPU), chosen for its elastomeric properties, and polyamide (PA12), selected for its rigidity. By incorporating recycled TR (up to 30 wt%), TPU/TR composites demonstrated suitable impact strength, while PA12/TR composites exhibited excellent mechanical properties even at higher TR loadings [242].

Upcycling is the process of transforming waste materials or unwanted products into new materials or products perceived to be of higher quality, often with added environmental or functional value [243,244]. The research applied the concept of upcycling to transform nylon powder residues from the SLS process into filament suitable for FDM 3D printing, giving new purpose to waste materials and promoting sustainability. Standard-dimension filaments were successfully produced by incorporating magnesium (Mg), resulting in materials with the necessary properties for 3D printing [245]. In this study [241], a thermoreversible Diels–Alder (DA) crosslinked network was successfully fabricated using selective laser sintering (SLS) as the 3d-printing method. The design freedom of the selective laser sintering (SLS) process for the proposed material was validated through the production of various models. Additionally, circularity was demonstrated by recycling printed parts, which maintained consistent resolution and preserved the chemical integrity of the material, enabling its reuse in future production.

Effective powder management is an important issue in selective laser sintering due to the limited reusability of most powders. Throughout the production process, the powder undergoes extended exposure to heat, which can deteriorate its properties and diminish its suitability for reuse in subsequent cycles. This degradation results in substantial amounts of waste powder, creating both economic and environmental challenges. To mitigate this issue, manufacturers are advised to establish return agreements with powder suppliers, facilitating the collection and remanufacturing of waste powder into fresh material for new production cycles. This strategy not only minimizes waste but also enhances resource efficiency and supports sustainability in additive manufacturing processes. Additionally, agreements can be made with distributor companies to produce fresh powder from waste powder [246], or with 3d-printer manufacturers to provide powder handling stations where used powder can be mixed with fresh powder for subsequent prints [247]. However, SLS printing is energy-intensive, as the entire printing chamber must be heated, consuming approximately 3.08 kWh—an order of magnitude higher than that of other 3d-printing technologies [248,249,250].

## 4. Conclusions and Future Perspectives

Plastic waste management has become a pressing environmental issue for humanity in recent decade. There is significant industrial and governmental interest in developing and implementing sustainable solutions to address waste management. This approach represents a critical step toward a green transition, promoting sustainable global practices across all fields of polymer processing and other industries. Achieving this transition requires a concerted and collaborative effort involving experts from industry, academia, governments, and individuals. Together, they must drive concrete measures to reduce unnecessary plastic usage and prioritize the selection of sustainable polymer sources and processing methods as part of an integrated, comprehensive solution. The integration of the circular economy concept with artificial intelligence (AI) can provide a synergistic approach to achieving a more sustainable and responsible future. Each processing method must develop tailored strategies and measures to address present and future challenges effectively. Additive Manufacturing (AM), in particular, offers significant advantages by requiring fewer resources during the research and development phase. Moreover, AM holds great potential in polymer recycling, enabling the reuse of various waste materials by transforming them into high-value polymeric products. The most effective strategy for achieving a sustainable injection molding process is to minimize waste at every stage. This begins with optimizing raw material usage by carefully considering factors such as sourcing, cost, and transportation. It continues through the processing steps, focusing on reducing energy consumption, processing time, and the use of human resources. Finally, sustainability efforts extend to the storage, transportation, and disposal of the final product, ensuring a comprehensive approach to waste reduction throughout the entire lifecycle. Choosing appropriate additives, such as plasticizers or lubricants, is an effective strategy to streamline processing and lower energy consumption. As environmental concerns intensify and the transition to sustainable materials gains momentum, the industry is advancing innovations to enhance the efficiency of extrusion technology. These efforts focus on developing energy-efficient systems that minimize energy usage and operational costs while supporting sustainable manufacturing practices. Sustainability can be incorporated into multiple stages of the extrusion process, starting with the selection of environmentally friendly materials and extending to process optimization to minimize waste and energy consumption. Additionally, it involves implementing practices that consider the entire product life cycle, including its environmental impact and strategies to mitigate it. These combined efforts play a crucial role in reducing the environmental footprint and improving the overall sustainability of polymer processing.

## Figures and Tables

**Table 1:** Commercially available sustainable materials for injection molding.

Product Name	Producer	Composition	Characteristics	Ref.
Terratek^®^ NFRP	Green dot^®^ BIOPLASTICS	Natural fiber-reinforced polymers with hemp, jute, sisal, bamboo, or flax	Lightweight and renewable content	[69]
Trifilon BioLite^®^ 240	Trifilon^TM^	PP and cellulose fibers	Reduces plastic usage by up to 30%	[70]
Futerro RENEW™ 801	Futerro	PLA	100% renewable and industrially compostable	[71]
REFINE^®^ PF3 434A	Automotive Performance Materials	Hemp fibers and PP	Reduce fossil fuel-based plastic content	[72]
Mater-Bi^®^	Novamont	Polymers made from renewable raw materials like starch, cellulose, vegetable oils and their combinations	Biodegradable and compostable options	[73]
BioBlend^®^ BC 27255	BioLogiQ	Plant-based Polysaccharide and PLA	100% biobased	[74]
CGT-ESR-XX-REV 3.7S	Competitive Green Technologies	Custom matrices with coffee chaff filler	100% industrial compostable	[75]
Ecovio^®^ IS1335	BASF	Polybutylene adipate terephthalate (PBAT) and PLA	Biodegradability	[76]

**Table 2:** Commercially available sustainable materials for extrusion.

Product Name	Composition	Characteristics	Application	Ref.
BiONext 102	PLA and CaCO_3_	Completely degradable within 12 months	Plastic straws	[137]
Terratek^®^ SC	Starch-polymer composites	Made from up to 65% renewable material	Profile extruded articles	[138]
Ingeo™ Biopolymer 2003D	PLA	Derived from annually renewable resources	Fresh food packaging	[139]
Nodax^®^ 2513	Polyhydroxyalkanoates (PHAs)	100% renewable, biobased, and biodegradable in marine, freshwater, soil, home and industrial compost, and via anaerobic digestion	Profile extrusion, drinking straws	[140]
Futerro RENEW™ 801	PLA	100% biodegradable in industrial composters	Packaging	[71]
BioBlend^®^ XD 25250	50% plant-based Polysaccharide and 50% polypropylene	Reduce both fossil fuel-based plastic content and greenhouse gas generation	Sheets	[141]
I am green™ bio-based polyethylene	Bio-based polyethylene from Brazilian sugarcane ethanol	Renewable alternative to fossil polyethylene	Tube extrusion	[142]
Natur-Tec^®^ BF5002	PLA	Up to 75% biobased and 100% compostable,	Compostable zippers for bags via profile extrusion	[143]
Biodolomer^®^ E 900328	Polybutylene adipate terephthalate (PBAT), PLA and minerals	Compostability and biodegradability	Thin tube, pipe and straws	[144]

**Table 3:** Commercially available filaments in FDM as sustainable alternatives.

Product Name	Specifications Outlined by the Manufacturer	Ref.
allPHA NATURAL	100% biodegradable 100% biobased without microplastics	[184]
0rCA^®^	100% recycled material composed of recycled marine Nylon (PA6) and carbon fiber (10%)	[185]
COMPOST3D^®^	made from natural raw materials 100% compostable	[186]
Wound Up—Coffee Filament	biobased made using waste byproducts from coffee and PLA	[187]
Algae Based PLA	5–10% algae content, derived from discarded post-industrial materials Algae increase the biodegradability	[188]
Wood Bamboo	real wood fiber makes about 30% of the material. Unequalled wood grain and texture	[189]
FL600EVA-BIO	bio-based ethylene vinyl acetate (EVA) filament, derived from raw sugarcane	[190]
FL600R	a recycled PE/PP blend containing over 90% of the material is obtained sustainably.	[191]
Green PLA	recycled content: 87% packaging and spools of cardboard that are 100% recyclable	[192]
